# A novel fully covered double-bump stent for staple line leaks after bariatric surgery: a retrospective analysis

**DOI:** 10.1007/s00464-018-6034-2

**Published:** 2018-01-17

**Authors:** Thomas C. C. Boerlage, Gerardus P. M. Houben, Marcel J. M. Groenen, Klaas van der Linde, Arnold W. J. M. van de Laar, Marloes Emous, Paul Fockens, Rogier P. Voermans

**Affiliations:** 1 Department of Internal Medicine, MC Slotervaart, Louwesweg 6, 1066 EC Amsterdam, The Netherlands; 2 Department of Gastroenterology & Hepatology, Academic Medical Center, Amsterdam, The Netherlands; 3 Department of Gastroenterology & Hepatology, MC Slotervaart, Amsterdam, The Netherlands; 4 Department of Gastroenterology & Hepatology, Rijnstate ziekenhuis, Arnhem, The Netherlands; 5 Department of Gastroenterology & Hepatology, MC Leeuwarden, Leeuwarden, The Netherlands; 6 Department of Surgery, MC Slotervaart, Amsterdam, The Netherlands; 7 Department of Surgery, MC Leeuwarden, Leeuwarden, The Netherlands

**Keywords:** Bariatric surgery, Roux-en-Y gastric bypass, Sleeve gastrectomy, Staple line leakage, Stent

## Abstract

**Background:**

Staple line leakage after bariatric surgery can be treated by endoscopic placement of a self-expandable stent. The success rate of stent placement is generally high, but migration is a frequent adverse event that hampers successful treatment. The Niti-S Beta stent is a fully covered double-bump stent that was specifically designed to prevent migration. This study aimed to evaluate the effectiveness and adverse event rate of the Niti-S Beta stent.

**Methods:**

A retrospective study was performed in three high-volume bariatric centers. All consecutive patients between 2009 and 2016 who underwent placement of a Beta stent for staple line leakage were included. Primary outcome was resolution of the leakage; secondary outcome was the adverse event rate including migration.

**Results:**

Thirty-eight patients were included. Twenty-five (66%) had resolution of the leakage. Success rate was higher in patients who were treated with implantation of a Beta stent as initial treatment (100%) than in patients who were treated with a stent after revisional surgery had failed (55%, *p* = 0.013). Migration occurred in 12 patients (32%). There were two severe adverse events requiring surgical intervention, including a bleeding from an aorto-esophageal fistula.

**Conclusions:**

The success rate and the migration rate of the Beta stent seem comparable to other stents in this retrospective study. Despite the novel double-bump structure of the stent, the migration rate does not seem to be decreased.

Bariatric surgery is the most effective long-term treatment for morbid obesity [1]. Laparoscopic Roux-en-Y gastric bypass (LRYGB) and laparoscopic sleeve gastrectomy (LSG) are the most commonly performed bariatric procedures worldwide [2]. Staple line leakage is a severe adverse event of both LRYGB and LSG with a prevalence of 1–2% [3, 4]. Revisional surgery (surgical repair of the staple line) is often necessary, although conservative management consisting of abscess drainage, antibiotics and nil per os in combination with a nasojejunal feeding tube is sometimes sufficient [5]. Endoscopically placed self-expandable stents can be an alternative to surgery in selected cases when there is relatively limited leakage, or when leakage persists despite revisional surgery. Stent placement is effective in more than 50% of these selected cases [6]. The most frequent adverse event of stent placement in patients after bariatric surgery is migration with a prevalence varying from 4.8 to 67%, depending on stent type and indication for placement [6–8].

The Niti-S Beta™ stent (TaeWoong medical, South Korea) is an over-the-wire nitinol stent, which is specifically designed for the treatment of staple line leakage after bariatric surgery. It has a flange at the proximal end and the proximal part of the stent is thickened by means of a silicone-covered double stent layer, creating a double-bump (Fig. 1). It is stated that the double-bump prevents migration by increasing the radial pressure, but there are no clinical or experimental data supporting this. The present study was designed to evaluate the success and adverse event rate of the Niti-S Beta stent.

**Fig. 1:**
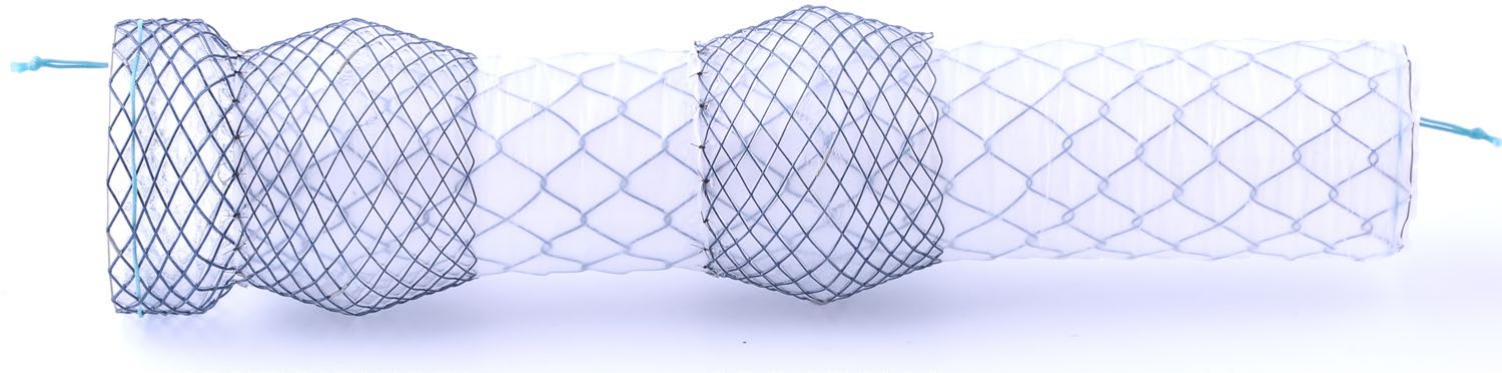
The Niti-S Beta stent

## Methods

A retrospective study was performed in three high-volume bariatric centers (each > 800 bariatric interventions annually) in the Netherlands. The endoscopy database in each centre was searched for all consecutive patients who underwent placement of a Niti-S Beta stent for staple line leakage after bariatric surgery. The medical ethics committee certified that formal ethical review was not necessary for this study.

### Stent procedure

The Niti-S Beta stent is a self-expandable fully covered double-bump metal stent with radiopaque markers at both ends. All stents were placed by endoscopists with experience in stent placement. The indication and timing of stent placement and the type and size of the stent were at the discretion of the local surgeon and endoscopist. The length of the stents ranged from 100 to 180 mm and the inner diameter from 20 to 28 mm. The location and size of the leak were determined endoscopically and marked with an external radio-opaque marker if preferred by the endoscopist. Next, a guidewire was placed through the scope in the duodenum or proximal jejunum. The scope was removed and the delivery device (20 to 22 Fr, length 70 cm) was introduced over the guidewire. The stent was placed under fluoroscopic guidance and/or endoscopic guidance at the discretion of the endoscopist. The stent was positioned so that the leak was in between the two bumps. After placement, the location was checked by fluoroscopy and endoscopic visualization. If necessary the stent was relocated. The day after stent placement, an upper gastrointestinal series or oral methylene blue test was performed to assess potential persisting leakage. In general, oral intake was started after proof of the absence of significant leakage. Timing of removal of the stent was determined by the endoscopist in discussion with the surgeon. The stent was removed using an endoscopic forceps grasping the proximal or distal retrieval string. Imaging was only repeated in case of suspicion of persisting or recurrent leakage.

### Outcome measures

The primary outcome was resolution of the leakage. Resolution was defined as closure of the leak (confirmed by endoscopic visualization of the leak or radiographic imaging with oral contrast) within 1 week after stent removal without signs of a recurrent leak after 6 weeks of follow-up and without need for additional surgical or endoscopic intervention. Secondary outcomes were adverse events of stent placement. Predefined adverse events were migration, fixation complicating removal, stenosis, bleeding, and complaints of pain or dysphagia due to the stent and unresponsive to medical treatment. Migration was defined as dislocation of the stent requiring an additional procedure, either endoscopic or surgical, to remove or reposition the stent. Fixation was defined as overgrowth of tissue at the stent margins hindering endoscopic removal and necessitating an additional (endoscopic) procedure for removal. Stenosis was defined as a stricture requiring endoscopic dilatation. Bleeding was defined as symptomatic gastrointestinal hemorrhage requiring a therapeutic intervention.

### Statistical analysis

For the primary outcome, a per-patient analysis was performed. Adverse events were analyzed per-stent and per-patient. Data extracted included demographics, medical history and medication use, type of bariatric surgery, time to stent placement, concurrent interventions, stent size, duration of treatment, reason for stent removal, adverse events, and outcome. Descriptive statistics were used for all data, with mean and standard deviation (SD) in case of normal distribution, and median and range for non-normal distribution. For the main outcomes, proportions and percentages are shown. Several factors associated with success were hypothesized beforehand. The differences in success and adverse event rate between stents placed as initial treatment versus stents after failed revisional surgery, first versus consecutive stents, and LRYGB versus LSG, were calculated with the Chi-square test. Potential influence of time between initial surgery and stent implantation on successful resolution was checked using the Mann–Whitney *U* test.

## Results

Thirty-eight patients were included, who underwent 50 stent placements in total. The first stent was placed in October 2009. The supplier of the stent (Prion Medical BV) confirmed that, from October 2009 to March 2016, 50 stents were provided to the participating centers. Thus, all consecutive stent placements during the entire study period have been included. Patient characteristics are shown in Table 1. Patients had a mean age of 45 and a mean BMI of 43.0 kg/m^2^. Twenty-nine patients (76%) were female. All but 3 patients underwent percutaneous abscess drainage.

**Table 1:** Characteristics of the included patients (*N* = 38)

Patient characteristics	
Age Mean (SD)	45.4 (10.0)
BMI preoperative Mean (SD)	43.0 (7.7)
Weight preoperative in kilograms Mean (SD)	125.1 (29.0)
Sex (female) *N* (%)	29 (76%)
Smoking *N* (%)	7 (18%)
Previous abdominal surgery *N* (%)	27 (71%)
Type of surgery *N* (%)	
Primary LRYGB	12 (32%)
Revisional LRYGB	10 (26%)
Modified LRYGB	1 (3%)
Primary LSG	12 (32%)
Revisional LSG	1 (3%)
Omega-loop Gastric Bypass	1 (3%)
SADI (revision after LSG)	1 (3%)

*BMI* Body Mass Index, *LRYGB* laparoscopic Roux-en-Y gastric bypass, *LSG* laparoscopic sleeve gastrectomy, *SADI* single anastomosis duodeno-ileal bypass

### Stent placement

In 9 patients (24%), the initial stent was placed as primary treatment for limited staple line leakage, a median of 20 days (range 0–152 days) after bariatric surgery (group 1). In 8 of these patients, a stent was placed after initial conservative treatment was not successful. In one patient, staple line leakage developed during the initial bariatric surgery and was treated with stent placement immediately during surgery. In 29 cases (76%), the stent was placed as a second treatment after revisional surgery had failed to resolve the leakage (group 2). This included 3 patients who underwent stent placement concurrently with revisional surgery which failed to control the leak. The median time between initial bariatric surgery and placement of the first stent in these cases was 14 days (range 2–735 days).

### Outcome

#### Success rate

In 25 out of 38 patients (66%), leakage was successfully treated with stent placement, including 14 out of 23 LRYGB patients (61%) and 9 out of 13 patients with LSG (69%, *p* = 0.616). One patient was lost to follow-up with a stent still in place. One patient died while the stent was in situ. Cause of death was persistent leakage in combination with pre-existing decompensated liver cirrhosis. These two stent placements were classified as unsuccessful. Follow-up data of at least 6 weeks after stent removal were available for the remaining 36 patients.

Patients who were treated with implantation of a Beta stent as initial treatment (group 1) had a higher chance of successful leak resolution (9 out of 9 cases, 100%) than patients who were treated with a stent after revisional surgery had failed (group 2; 16 out of 29 cases, 55%, *p* = 0.013). Success rate was not related to the median time between initial bariatric surgery and placement of the first Beta stent, which was 16 days in the successful group versus 12 days in the unsuccessful group (*p* = 0.973).

#### Number of stents required

**Fig. 2:**
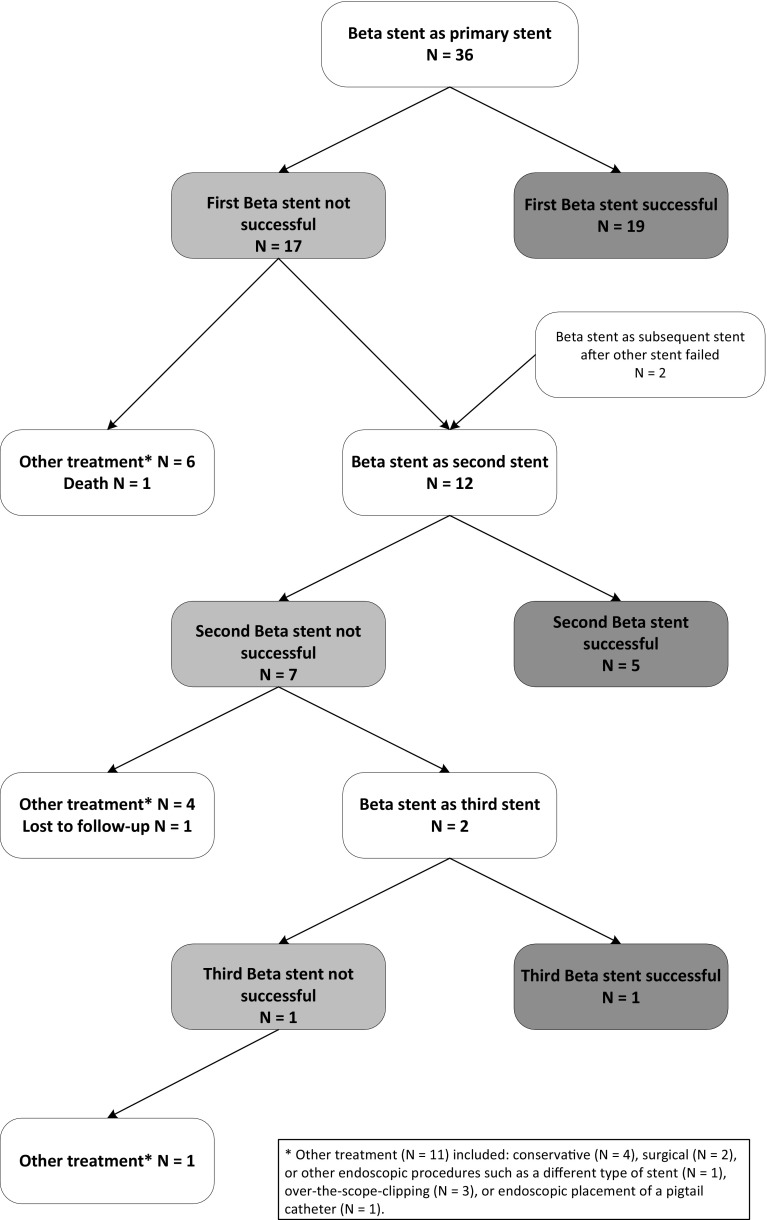
Flowchart of stent placements

In 19 out of 38 cases (50%), one stent was sufficient, including all 9 patients (100%) in group 1 and 10 out of 29 patients (35%) in group 2. Of the remaining 19 patients in group 2, 6 patients received another treatment after the first stent failed and one patient died. In 12 patients, the first stent was removed and a second Beta stent was placed because of persistent leakage after a median of 20 days after first stent placement. Two of these patients had initially been treated with a different type of stent and received a Beta stent as secondary treatment. Five out of 12 patients (42%) showed resolution of the leakage after the second stent. Four patients received another treatment after the second stent failed; one patient was lost to follow-up, and 2 patients received a third Beta stent, which was successful in one case (see Fig. 2). The chance of success of a consecutive stent did not differ from success after the first stent (6 out of 14 vs. 19 out of 36, *p* = 0.529).

#### Other treatment after stent failure

In total, 11 patients had no resolution of the leakage after one or multiple stent placements. These patients were successfully treated with revisional surgery (*n* = 2), conservative treatment (*n* = 4) or other endoscopic interventions such as a different type of stent (*n* = 1), over-the-scope-clipping (*n* = 3) or endoscopic placement of a pigtail catheter (*n* = 1).

#### Stent removal

Stents were left in place for a mean time of 27.7 days (SD 14.3). The stent was removed according to plan in 29 cases after a mean time of 36.0 days (SD 10.0). In 19 cases, the stent was removed prematurely after a mean time of 15.0 days (SD 9.8), because of migration without options for repositioning (*n* = 8), complaints of dysphagia and pain unresponsive to medical treatment (*n* = 7), persistent leakage (*n* = 3), and hemorrhage caused by the stent (*n* = 1). All stents were endoscopically removed without problems.

### Migration and other adverse events

In total, 14 patients (37%) experienced one or more adverse events of stent placement. Migration occurred in 13 stents (26%) in 12 different patients (32%), including 8 out of 31 stents in LRYGB patients (26%) and 3 out of 16 stents in patients with LSG (19%, *p* = 0.588). In 5 cases, it was possible to reposition the migrated stent endoscopically. In the remaining 8 cases, repositioning was not possible and the stent was removed. In one of these patients, the stent had migrated through the leak to the retroperitoneal area and had to be removed surgically. One patient (3%) required balloon dilatation for a benign stenosis 4 months after stent removal. There was one case of hemorrhage in this series, due to an aorto-esophageal fistula caused by mechanical pressure of the stent. The patient presented with hematemesis 3 weeks after placement of a (second) Beta stent for staple line leakage. An aorto-esophageal fistula was diagnosed at the site of the bump and treated with placement of an endovascular covered aortic stent. This case has earlier been published as a case report [9].

## Discussion

Endoscopic placement of a self-expandable stent is an increasingly used treatment option for staple line leakage after bariatric surgery [6, 8, 10–12]. This is the first large series describing the effectiveness and adverse event rate of the Niti-S Beta stent, a fully covered double-bump metal stent.

This study shows that staple line leakage after bariatric surgery can be effectively treated with the Beta stent in 66% of cases. In a recently published series, with 10 patients who had staple line leakage after LSG, the success rate of the Beta stent was 80% [13]. This success rate seems comparable to the results of other stents in previous studies, which ranges from 62 to 96% [7, 8, 10–12, 14–19]. However, comparison is hampered because all studies, including this study, are retrospective in nature and therefore subject to selection bias. In addition, there are differences in indication, definition of success and stent type between studies. For example, in the study describing the highest success rate (96%), a partially covered metal stent was placed in all patients with staple line leakage, including small leaks that might also have closed with conservative management only [15]. The definition of success in our study was stricter than in some other studies. Four patients who had resolution of the leakage more than 1 week after stent removal, but without additional interventions, were not considered successful in this study. If these patients had been defined as successful, as in several other studies, the success rate would have been higher (76%).

The most striking factor associated with success in this study was the difference between stents placed as initial treatment (group 1) versus those placed after failed revisional surgery (group 2). In group 1, the success rate was 100%, compared to 55% success rate in group 2. This result raises the question whether immediate stent placement should be considered in all patients with staple line leakage [15]. However, it must be underlined that the difference between the two groups in this study is subject to considerable bias. Stent placement was mostly the initial treatment in patients with only minor leakage. In contrast, leaks that persist after revisional surgery are generally larger and more difficult to close. Therefore, the chance of successful stent treatment in these cases may be lower.

Time between initial bariatric surgery and stent placement was not a predictor of success in this study. This is in contrast to previous studies, where stent placement early after surgery increased the chance of successful leak resolution [10, 14]. This variation between studies is most likely due to selection bias, with differences in indication and timing of stent placement between studies. Whether early stent placement does or does not increase the success rate should be subject of further prospective research.

In previous studies, placement of a consecutive stent when the first stent failed to resolve the leakage was feasible and had a considerable success rate [6]. The chance of success of a consecutive stent did also not differ from the first stent in our series. This confirms that placement of a consecutive stent should be considered when the first stent has failed to resolve the leakage.

The most frequent adverse event of stent placement is migration [6–8]. Despite the novel double-bump structure of the Beta stent, which has specifically been designed to prevent migration, migration occurred in 32% of patients (26% of stents) in this study. This seems comparable to the results of other fully covered stents, with a migration rate of 18–67% in previous studies [8, 11, 16, 17, 19, 20]. Partially covered stents are less prone to migration with a migration rate of 5–15% [7, 10, 14, 15, 18]. However, these stents are often difficult to remove [7]. Several options to prevent migration of fully covered stents have been described. Two studies describe fixation of the stent by endoscopic clipping, but only a small number of patients with staple line leakage was included [21, 22]. Some authors advocate suturing of the stent to the esophageal wall to prevent migration. However, migration rate was still 33% in one study [23].

In this study, there was one death unrelated to the procedure and no stent-related deaths. However, there was one life-threatening adverse event: hemorrhage from an aorto-esophageal fistula caused by mechanical pressure of the stent. In previous studies, mortality was described as well, most often not stent-related [5, 7, 8, 24]. Stent-related deaths, due to perforation or bleeding, were described in two studies [10, 19]. In one study, a fatal bleeding was caused by the mechanical pressure of the stent, which caused the bleeding in this study too [19]. Therefore, this adverse event does not seem to be unique for the Beta stent. However, the double-bump structure of the stent leads to increased mechanical pressure at the level of the two proximal bumps on the esophagus, which may increase the risk of eroding adverse events such as happened in this case.

Alternative endoscopic treatment options for staple line leakage include over-the-scope clipping, overstitching of the leak in case of small leaks, endoluminal vacuum therapy, or drainage with a pigtail [25–28]. However, due to the small number of patients in these studies and the lack of prospective studies and studies comparing different treatment options, it is yet unclear which treatment option has the highest chance of success.

The main limitation of this study is the retrospective design, which has led to a selection bias. The multicenter design is both a strength and a limitation of this study. There existed small differences in the indication for stent placement between the participating centers. However, the technique for placement was comparable between centers.

In conclusion, placement of the Niti-S Beta stent for staple line leakage after bariatric surgery is feasible and the results seem to be similar to other stents. This study does not support the statement that the double-bump structure prevents migration. However, prospective data are needed.

## References

[1] Colquitt JL, Pickett K, Loveman E, Frampton GK (2014). Surgery for weight loss in adults. Cochrane Database Syst Rev.

[2] Angrisani L, Santonicola A, Iovino P, Formisano G, Buchwald H, Scopinaro N (2015). Bariatric Surgery Worldwide 2013. Obes Surg.

[3] Anderin C, Gustafsson UO, Heijbel N, Thorell A (2015). Weight loss before bariatric surgery and postoperative complications: data from the Scandinavian Obesity Registry (SOReg). Ann Surg.

[4] Stroh C, Kockerling F, Volker L, Frank B, Stefanie W, Christian K, Christiane B, Thomas M (2016). Results of more than 11,800 sleeve gastrectomies: data analysis of the german bariatric surgery registry. Ann Surg.

[5] Gonzalez R, Sarr MG, Smith CD, Baghai M, Kendrick M, Szomstein S, Rosenthal R, Murr MM (2007). Diagnosis and contemporary management of anastomotic leaks after gastric bypass for obesity. J Am Coll Surg.

[6] Puli SR, Spofford IS, Thompson CC (2012). Use of self-expandable stents in the treatment of bariatric surgery leaks: a systematic review and meta-analysis. Gastrointest Endosc.

[7] Eisendrath P, Cremer M, Himpens J, Cadiere GB, Le Moine O, Deviere J (2007). Endotherapy including temporary stenting of fistulas of the upper gastrointestinal tract after laparoscopic bariatric surgery. Endoscopy.

[8] van Wezenbeek MR, de Milliano MM, Nienhuijs SW, Friederich P, Gilissen LP (2016). A specifically designed stent for anastomotic leaks after bariatric surgery: experiences in a Tertiary Referral Hospital. Obes Surg.

[9] Boerlage TC, Hermanides HS, Moes DE, Tan IL, Houben GM, Acherman YI (2016). Aorto-oesophageal fistula after oesophageal stent placement in a patient with a Roux-en-Y gastric bypass. Ann R Coll Surg Engl.

[10] Murino A, Arvanitakis M, Le Moine O, Blero D, Deviere J, Eisendrath P (2015). Effectiveness of endoscopic management using self-expandable metal stents in a large cohort of patients with post-bariatric leaks. Obes Surg.

[11] Quezada N, Maiz C, Daroch D, Funke R, Sharp A, Boza C, Pimentel F (2015). Effect of early use of covered self-expandable endoscopic stent on the treatment of postoperative stapler line leaks. Obes Surg.

[12] Southwell T, Lim TH, Ogra R (2016). Endoscopic therapy for treatment of staple line leaks post-laparoscopic sleeve gastrectomy (LSG): experience from a Large Bariatric Surgery Centre in New Zealand. Obes Surg.

[13] Tringali A, Bove V, Perri V, Landi R, Familiari P, Boskoski I, Costamagna G (2017). Endoscopic treatment of post-laparoscopic sleeve gastrectomy leaks using a specifically designed metal stent. Endoscopy.

[14] Alazmi W, Al-Sabah S, Ali DA, Almazeedi S (2014). Treating sleeve gastrectomy leak with endoscopic stenting: the Kuwaiti experience and review of recent literature. Surg Endosc.

[15] El Mourad H, Himpens J, Verhofstadt J (2013). Stent treatment for fistula after obesity surgery: results in 47 consecutive patients. Surg Endosc.

[16] Fishman S, Shnell M, Gluck N, Meirsdorf S, Abu-Abeid S, Santo E (2015). Use of sleeve-customized self-expandable metal stents for the treatment of staple-line leakage after laparoscopic sleeve gastrectomy. Gastrointest Endosc.

[17] Iqbal A, Miedema B, Ramaswamy A, Fearing N, de la Torre R, Pak Y, Stephen C, Thaler K (2011). Long-term outcome after endoscopic stent therapy for complications after bariatric surgery. Surg Endosc.

[18] Salinas A, Baptista A, Santiago E, Antor M, Salinas H (2006). Self-expandable metal stents to treat gastric leaks. Surg Obes Relat Dis.

[19] Shehab HM, Hakky SM, Gawdat KA (2016). An endoscopic strategy combining mega stents and over-the-scope clips for the management of post-bariatric surgery leaks and fistulas (with video). Obes Surg.

[20] Freedman J, Jonas E, Naslund E, Nilsson H, Marsk R, Stockeld D (2013). Treatment of leaking gastrojejunostomy after gastric bypass surgery with special emphasis on stenting. Surg Obes Relat Dis.

[21] Irani S, Baron TH, Gluck M, Gan I, Ross AS, Kozarek RA (2014). Preventing migration of fully covered esophageal stents with an over-the-scope clip device (with videos). Gastrointest Endosc.

[22] Vanbiervliet G, Filippi J, Karimdjee BS, Venissac N, Iannelli A, Rahili A, Benizri E, Pop D, Staccini P, Tran A, Schneider S, Mouroux J, Gugenheim J, Benchimol D, Hebuterne X (2012). The role of clips in preventing migration of fully covered metallic esophageal stents: a pilot comparative study. Surg Endosc.

[23] Fujii LL, Bonin EA, Baron TH, Gostout CJ, Wong Kee Song LM (2013). Utility of an endoscopic suturing system for prevention of covered luminal stent migration in the upper GI tract. Gastrointest Endosc.

[24] Swinnen J, Eisendrath P, Rigaux J, Kahegeshe L, Lemmers A, Le Moine O, Deviere J (2011). Self-expandable metal stents for the treatment of benign upper GI leaks and perforations. Gastrointest Endosc.

[25] Cai JX, Khashab MA, Okolo PI, Kalloo AN, Kumbhari V (2014). Full-thickness endoscopic suturing of staple-line leaks following laparoscopic sleeve gastrectomy. Endoscopy.

[26] Donatelli G, Dumont JL, Dhumane P, Dritsas S, Tuszynski T, Vergeau BM, Meduri B (2017). Double pigtail stent insertion for healing of leaks following Roux-en-Y gastric bypass. Our experience (with videos). Obes Surg.

[27] Keren D, Eyal O, Sroka G, Rainis T, Raziel A, Sakran N, Goitein D, Matter I (2015). Over-the-scope clip (OTSC) system for sleeve gastrectomy leaks. Obes Surg.

[28] Leeds SG, Burdick JS (2016). Management of gastric leaks after sleeve gastrectomy with endoluminal vacuum (E-Vac) therapy. Surg Obes Relat Dis.

